# Acupuncture Reduces the Risk of Dysphagia in Stroke Patients: A Propensity Score-Matched Cohort Study

**DOI:** 10.3389/fnins.2021.791964

**Published:** 2022-01-06

**Authors:** Xuan Qiu, Xiao-Jie Yao, Sheng-Nan Han, Yun-Yun Wu, Zeng-Jian Ou, Tian-Shi Li, Hong Zhang

**Affiliations:** ^1^Clinical Medical College of Acupuncture, Moxibustion and Rehabilitation, Guangzhou University of Chinese Medicine, Guangzhou, China; ^2^First Affiliated Hospital of Guangzhou University of Chinese Medicine, Guangzhou, China

**Keywords:** acupuncture, stroke, dysphagia, cohort study, propensity score matching

## Abstract

**Background:** Post-stroke dysphagia (PSD) affects the quality of life in stroke patients, impairs their rehabilitation ability, and causes other complications following stroke. Currently, there is currently some understanding of PSD risk factors, but its protective factors remain largely unknown.

**Objective:** To analyze the effects of acupuncture (AP) on dysphagia in stroke patients and explore its potential as a preventive therapy.

**Methods:** Patients with a diagnosis of stroke from 2010 to 2019 were selected and followed until 2020, utilizing factors such as age, gender, stroke location, stroke type, and baseline comorbidity. To compare the incidence of dysphagia, equal numbers of stroke patients treated with and without AP (*n* = 1,809) were matched by 1:1 propensity scoring. The Cox proportional hazards model and Kaplan-Meier method were used to assess the risk of dysphagia as an outcome measure.

**Results:** The stroke patients treated with AP had a lower risk of dysphagia after adjusting for age, gender, stroke location, stroke type, and baseline comorbidity [adjusted hazard ratio (AHR) = 0.43, 95% confidence interval = 0.37–0.49] compared with those in the non-AP cohort. AP also decreased the risk of PSD among different gender groups. The risk ratios were AHR = 0.45 and AHR = 0.33 for males and females, respectively. AP also reduced the risk for PSD among different age groups. The risk ratios were AHR = 0.20, AHR = 0.37, AHR = 0.41, and AHR = 0.45 for the 18–39, 40–59, 60–79, and >80 years-old groups. Regarding stroke types (ischemic, hemorrhagic, and mixed type), patients treated with AP had a lower risk (AHR = 0.47, 0.28 and 0.17, respectively). With respect to stroke location, the risk of PSD in AP-treated patients was decreased regardless of location: brain stem (AHR = 0.41), diencephalon (AHR = 0.13), or multiple lesions (AHR = 0.40), the risk of PSD in AP-treated patients was decreased. For all baseline comorbidities, AP attenuated the risk of dysphagia. The cumulative incidence of dysphagia was remarkably lower in the AP group than in the non-AP group (log-rank test, *P* = 0.000).

**Limitations:** First, this was a single-center clinical retrospective study. Second, we did not classify the severity of stroke and dysphagia. Third, all data were extracted manually. Lastly, the sample size was relatively small. Thus, future studies with larger sample sizes are warranted to verify our findings.

**Conclusion:** Acupuncture treatment attenuates the risk of dysphagia in stroke patients. Future research should increase the sample size and elaborate further on the details of the AP protocol.

## Introduction

Stroke is the main cause of dysphagia (Jones et al., 2020), with an incidence rate of 30–40% (Bussell and González-Fernández, 2011; Meng et al., 2020). Dysphagia after stroke leads to many adverse outcomes. Long-term dysphagia after stroke leads to respiratory infections, malnutrition, disability, and death (González-Fernández et al., 2013). Swallowing dysfunction after a stroke patient is discharged from hospital is a sign of a poor prognosis after the first stroke (Souza et al., 2020). Dysphagia also prolongs the duration of hospital stay and increases the difficulty of care due to aspiration (Joundi et al., 2017) and the possibility of the patient being re-admitted to hospital (Cabré et al., 2014; Arnold et al., 2016). Early recognition of dysphagia can reduce undesirable results (Sivertsen et al., 2017). The most widely used clinical treatment of post-stroke dysphagia (PSD) is electrical stimulation-related swallowing therapy that includes surface neuromuscular electrical stimulation, transcranial magnetic stimulation, and surface neuromuscular electrical stimulation (Qin et al., 2020). However, high-quality evidence-based studies have reported that there are hidden dangers in the effectiveness and safety of swallowing therapy. Researchers realized that the early preventive treatment of PSD is very important (Andrade et al., 2017) and therefore there is a need to explore other effective treatments, especially preventive treatments and determine which are the most effective (Bath et al., 2018).

Neuroplasticity is crucial in the recovery of swallowing function (Barritt and Smithard, 2009). Studies have shown that drug therapy may be an effective as a preventive treatment for PSD by improving neurological functions; however, there is insufficient evidence on this possibility because of the small sample size in relevant research studies (Huang et al., 2018). Acupuncture (AP) has been shown to significantly improve the plasticity of nerves after a stroke, but studies used AP in combination with other treatments, such as combined swallowing training (Mao et al., 2016) or other rehabilitation training (Chen and Guo, 2018). Therefore, studies should analyze on the advantages of AP alone in the treatment of PSD. In 2018, the results of a meta-analysis published by Li et al. (2018) proved that AP alone had some advantages in improving dysphagia after stroke. However, the evidence-based quality of these studies was low (Li et al., 2018). Therefore, studies should analyze the intervention effect of AP on the onset of dysphagia after stroke, and to explore the advantages of AP from the perspective of treatment rather than the perspective of prevention. Predicting the occurrence of dysphagia can reduce its incidence and reduce its risk of other diseases. Few studies have reported on the prevention of dysphagia. Zhou et al. (2019) reported a scoring system for the early prediction of dysphagia, which was used to screen high-risk groups of dysphagia, but this method is not specific and required patients to have a certain ability to cooperate, resulting in poor overall adaptability. Also Perry et al. (2019) proposed to exercise patients’ cough reflex to identify the risk of dysphagia early and reduce its harm. However, this method requires clinicians for the first time to recognize whether the patient has a cough reflex, which leads to poor clinical availability. Currently, there is a lack of clinical retrospective studies on dysphagia after stroke or analyzing the advantages of reducing the occurrence of dysphagia after stroke from the perspective of prevention. Therefore, we performed a retrospective clinical study of AP to reduce dysphagia after stroke using a large sample size, and explored the feasibility and advantages of AP to prevent PSD.

## Materials and Methods

### Data Source

In this clinical retrospective study, the patient’s data were collected from the First Affiliated Hospital of Guangzhou University of Chinese Medicine (GUCM). Ethical approval for this study was obtained from the hospital’s institutional review board. The patients diagnosed with stroke were recruited at the First Affiliated Hospital of GUCM from 2010 to 2020. All clinical information was retrieved from the electronic medical record system. The diagnostic codes were in accordance the International Classification of Diseases, Tenth Revision, Clinical Modification (ICD-10-CM).

The inclusion criteria were as follows: (i) at least 18 years old or older; (ii) the diagnostic criteria of acute ischemic stroke are in line with the “Guidelines for the Diagnosis and Treatment of Acute Ischemic Cerebrovascular Diseases in China”: (1) acute onset; (2) focal neurological deficit or comprehensive neurological deficit; (3) with imaging of the responsible lesions or signs lasting for more than 24 h; (4) excluding non-vascular causes; (5) excluding cerebral hemorrhage by brain CT/MRI; the guidelines for cerebral hemorrhage comply with the Chinese guidelines for the diagnosis and treatment of cerebral hemorrhage: (1) acute onset; (2) focal neurological deficit symptoms (a few are general neurological deficits), often accompanied by headache, vomiting, elevated blood pressure, and varying degrees of consciousness disorders; (3) CT or MRI of the head shows hemorrhage; (4) excluding non-vascular brain causes; (iii) underwent a brain CT or MRI imaging; (iv) the clinical symptoms present within the first 24 h; (v) stroke was diagnosed prior to dysphagia; and (vi) multiple courses of AP treatment.

### Research Participants

**FIGURE 1:**
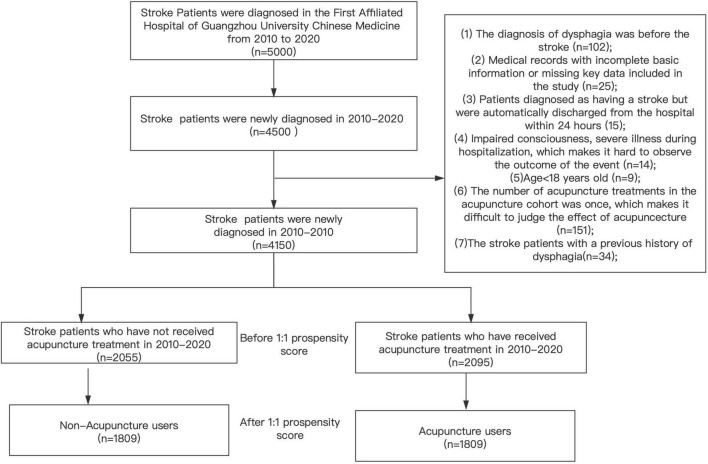
Process of study subjects.

All stroke patients (*n* = 5,000, ICD-10-CM: I61, I62, I63, I64, I65, and I66) were recruited at the First Affiliated Hospital of GUCM from January 1, 2010 to December 31, 2020. Among them, 4,500 patients were newly diagnosed with stroke. The exclusion criteria were as follows: (i) dysphagia occurred before stroke (ICD-10-CM:G52, R47, including I61, I62, I63, I64, I65, and I66); (ii) baseline information was not found in the medical record system and/or missing data; (iii) patients had a diagnosis of stroke, but were discharged from the hospital within 24 h and whose outcomes were not examined; (iv) patients experienced severe illness during hospitalization, and no outcomes were observed; (v) less than 18 years of age; (vi) only a single course of AP treatment and its impact was ascertained; (vii) stroke patients with a prior history of dysphagia. As a result, 2,095 stroke patients received AP treatment among the 4,150 patients initially enrolled in the study. The 1:1 propensity score matching method was used to match the two cohorts based on age, gender, stroke location, stroke type, and baseline comorbidity. The matching value was fixed at 0.0001, and 3,618 patients were matched. Overall, 1,809 patients were included in the AP or non-AP cohorts each (Figure 1).

### Covariate Assessment

In our study, the following covariate factors were included in the analysis

(i) Basic patient information, including name, medical record ID, gender, age, and date of admission;
(ii) information related to stroke, including time of first stroke, stroke type (ischemic stroke, or hemorrhagic stroke, or mixed), stroke location (frontal lobe, parietal lobe, occipital lobe, temporal lobe, radiation crown, brain stem, diencephalon, or multiple sites);
(iii) information related to the AP procedure that included the AP exposure factor for which observation was divided into the AP cohort group, non-AP cohort group, and the follow-up that was recorded in the AP cohort;
(iv) baseline diseases, including any previous hypertension (ICD-10-CM:I10), diabetes (ICD-10-CM:E10-14), hyperlipidemia (ICD-10-CM:E78), mental disorders (ICD-10-CM:F31, F32, F34, and F41), repeated strokes, dementia (ICD-10-CM:F00-03), Parkinson’s disease (ICD-10-CM:G20-G22), brain injury (ICD-10-CM:S01-S09), Lung infection (ICD-10-CM:J12-J19), and carotid atherosclerosis (ICD-10-CM: I70.8) were recorded.

### Outcome Measurements

We considered dysphagia to be an end-point event in the two stroke cohorts after the end of the follow-up time. The date for the AP treatment after the first diagnosis of stroke was defined as the index day. The main goal of the study was to compare the risk of dysphagia with the cohorts that did and did not receive AP. A determination that dysphagia was present primarily depended on: (1) direct diagnosis: diagnosis of dysphagia after diagnosis of stroke; and (2) diagnosis through symptoms and physical examination: according to the guidelines (Malagelada et al., 2014), the symptoms related to dysphagia were listed in the patient’s medical record: indwelling stomach tube, semi-liquid diet, or liquid diet, prevention of aspiration, swallowing training, and drinking test. If one of the two diagnostic methods was listed, we judged that the patient to have dysphagia.

### Statistical Analysis

To assess the differences in the baseline features between the AP and non-AP groups, the continuous and categorical variables were analyzed using the Student’s *t*-test and chi-square test, respectively. The hazard ratio (HR) with 95% confidence interval (CI) was determined by the Cox proportional hazard regression. Differences in the cumulative incidence of dysphagia between the two groups during the follow-up period were compared using the log-rank test and Kaplan-Meier method. The level of statistical significance was set at *p* < 0.05. All statistical tests were conducted using the SPSS v23.0 software.

## Results

As shown in Table 1, there were significantly more men than females in the two groups. The mean age was 65 years, and the 60–79 age group had the most stroke patients, accounting for 54% of all stroke patients. The most common type of stroke was ischemic stroke in both cohorts, accounting for 86.1% of all stroke patients. Most of the stroke patients had multiple lesion sites, accounting for 75.6% of all stroke patients. The average (median) follow-up period of dysphagia in the AP and non-AP cohorts was 335 (55) days and 184 (28) days, respectively. In addition, no significant differences in age, gender, stroke location, stroke type, or baseline comorbidity were found between the two groups after matching (*P* > 0.05).

**TABLE 1:** Characteristics of stroke patie.

Variable	Acupuncture treatment				*p-*value*
	No (*n* = 1,809)		Yes (*n* = 1,809)		
	n	%	n	%	
**Gender**					0.519
Women	579	32	561	31	
Men	1,230	68	1,248	69	
**Age group**					
18–39	42	2.3	42	2.3	1.000
40–59	538	29.7	538	29.7	
60–79	976	54	976	54	
≥80	253	14	253	14	
Mean ± SD (years)^†^	65.24	65.46			
	12.92	12.63			
**Stroke type**					1.000
Ischemic stroke	1,558	86.1	1,558	86.1	
Hemorrhagic stroke	186	10.3	186	10.3	
Mixed types of stroke	65	3.6	65	3.6	
**Location of the stroke**					1.000
Frontal lobe	23	1.3	23	1.3	
Parietal lobe	13	0.7	13	0.7	
Occipital lobe	16	0.9	16	0.9	
Temporal lobe	9	0.5	9	0.5	
Radiation crown	246	13.6	246	13.6	
Brain stem	91	5	91	5	
Diencephalon	44	2.4	44	2.4	
Multiple lesions	1,367	75.6	1,367	75.6	
**Baseline disease**					
Hypertension	941	52.0	941	33.5	1.000
Diabetes	606	52.0	576	31.8	0.288
Hyperlipidemia	555	30.7	544	30.1	0.691
Mental disorder	154	8.5	174	9.6	0.247
Repeated strokes	309	17.1	312	17.2	0.895
Dementia	225	12.4	244	13.5	0.347
Parkinson’s	122	6.7	122	6.7	1.000
Brain injury	110	6.1	105	5.8	0.725
Lung infection	174	9.6	168	9.3	0.733
Carotid atherosclerosis	591	32.7	638	34	0.099
Duration between dysphagia date and index, days (mean, median)	184 (28)		335 (55)		
Time from first acupuncture to stroke days (mean, median)	–	76 (8)			

*The average (median) follow-up period of dysphagia in the acupuncture cohort and non-acupuncture cohort were 335 (55) days and 184 (28) days, respectively. In the acupuncture cohort, the average (median) was 76 (8) days, respectively, for index days.*

*Chi-squared test.

*^†^t-test.*

*Repeated strokes are defined as more than one stroke.*

*Mental disorder includes anxiety and depression.*

*Mixed type of stroke includes ischemic type and hemorrhagic stroke.*

Dysphagia occurred in 918 patients with stroke. We employed crude HR (CHR) and adjusted HR (AHR) to assess the impact of various factors on dysphagia. As presented in Table 2, patients receiving AP had a significantly lower risk of dysphagia compared with patients not receiving AP (CHR = 0.45, *P* = 0.000, AHR = 0.43, *P* = 0.000) before and after the adjustment. Considering gender, no matter before or after adjustment, no obvious difference in dysphagia risk was found between males and females before or after adjustment (CHR = 1.03, *P* = 0.663, AHR = 1.04, *P* = 0.606). Regarding different age groups exhibited no discernible difference in risk before adjustment (*P* > 0.05); however, after adjustment, the 60–79-year-old group had a lower risk (AHR = 0.63, *P* = 0.044). Compared with ischemic stroke, hemorrhagic stroke and mixed type stroke showed higher risk before and after adjusting for the influence of other factors (hemorrhagic stroke: CHR = 1.48, *P* = 0.000, AHR = 1.55, *P* = 0.000; mixed type stroke: CHR = 1.67, *P* = 0.001, AHR = 1.47, *P* = 0.011). Regarding the stroke location, multiple locations (>2 lesion sites) of stroke had a higher risk before or after adjustment for the impact of other confounding covariates (CHR = 4.29, *P* = 0.012, AHR = 3.39, *P* = 0.035), whereas other stroke locations (brain stem, diencephalon, frontal lobe, occipital lobe, parietal lobe, radiation crown, or temporal lobe) showed no significant differences (*P* > 0.05).

**TABLE 2:** Cox model with hazard ratios and 95% confidence intervals of each covariate that developed into dysphagia after stroke.

Variable	No. of event	Crude*			Adjusted^†^		
	(*n* = 918)	HR	(95% CI)	*p*-value	HR	(95% CI)	*p*-value
**Acupuncture**							
No	519	1.00	Reference		1.00	Reference	
Yes	399	0.45	(0.39–0.51)	0.000	0.43	(0.37–0.49)	0.000
**Gender**							
Women	292	1.03	(0.90–1.19)	0.663	1.04	(0.90–1.20)	0.606
Men	626	1.00	Reference		1.00	Reference	
**Age group**							
18–39	23	1.39	(0.89–2.15)	0.145	0.83	(0.54–1.28)	0.403
40–59	247	1.07	(0.88–1.32)	0.495	0.68	(0.44–1.04)	0.071
60–79	500	0.98	(0.82–1.18)	0.830	0.63	(0.40–0.99)	0.044
≥80	148	1.00	Reference		1.00	Reference	
**Stroke type**							
Ischemic stroke	751	1.00	Reference		1.00	Reference	
Hemorrhagic stroke	119	1.48	(1.22–1.80)	0.000	1.55	(1.26–1.89)	0.000
Both types of stroke	48	1.67	(1.25–2.24)	0.001	1.47	(1.09–1.97)	0.011
**Location of the stroke**							
Frontal lobe	3	1.00	Reference		1.00	Reference	
Parietal lobe	3	2.31	(0.47–11.43)	0.307	1.65	(0.33–8.24)	0.544
Occipital lobe	5	2.17	(0.52–9.08)	0.289	2.22	(0.53–9.30)	0.276
Temporal lobe	2	3.00	(0.50–17.91)	0.230	3.15	(0.52–18.88)	0.210
Radiation crown	51	1.72	(0.54–5.52)	0.359	1.56	(0.49–5.01)	0.455
Brain stem	29	2.68	(0.82–8.80)	0.104	2.05	(0.62–6.75)	0.238
Diencephalon	17	2.44	(0.72–8.34)	0.154	2.33	(0.68–7.98)	0.177
Multiple lesions	808	4.29	(1.38–13.32)	0.012	3.39	(1.09–10.56)	0.035
**Baseline disease**							
Hypertension	594	1.71	(1.50–1.96)	0.000	1.51	(1.31–1.74)	0.000
Diabetes	424	1.56	(1.37–1.78)	0.000	1.32	(1.15–1.51)	0.000
Hyperlipidemia	413	1.83	(1.60–2.08)	0.000	1.53	(1.34–1.75)	0.000
Mental disorder	188	1.99	(1.70–2.34)	0.000	1.55	(1.31–1.84)	0.000
Repeated strokes	368	1.32	(1.15–1.51)	0.000	0.92	(0.79–1.06)	0.250
Dementia	273	2.12	(1.84–2.45)	0.000	1.62	(1.38–1.91)	0.000
Parkinson’s	123	1.70	(1.41–2.06)	0.000	0.99	(0.80–1.23)	0.994
Brain injury	104	1.85	(1.51–2.27)	0.000	1.40	(1.13–1.74)	0.002
Lung infection	130	1.34	(1.11–1.61)	0.002	1.22	(1.01–1.48)	0.044
Carotid atherosclerosis	389	1.33	(1.16–1.51)	0.000	1.17	(1.02–1.34)	0.025

*Crude HR: relative hazard ratios.*

*Adjusted HR: adjusted hazard ratio: mutually adjusted for Age, gender, stroke type, stroke site, and baseline diseases: hypertension, diabetes, hyperlipidemia, mental disorder, repeated strokes, rementia, Parkinson’s, brain Injury, lung infection in Cox proportional hazards regression.*

*Chi-squared test.

*^†^t-test.*

Among the baseline comorbidities, diabetes (AHR = 1.32, *P* = 0.000), hyperlipidemia (AHR = 1.53, *P* = 0.000), hypertension (AHR = 1.51, *P* = 0.000), mental disorder (AHR = 1.55, *P* = 0.000), rementia (AHR = 1.62, *P* = 0.000), brain injury (AHR = 1.40, *P* = 0.002), lung infection (AHR = 1.22, *P* = 0.044), and carotid arteriosclerosis (AHR = 1.17, *P* = 0.025) could increased the risk for dysphagia in stroke patients. However, repeated strokes (CHR = 1.32, *P* = 0.000, AHR = 0.92, *P* = 0.250) and Parkinson’s disease (CHR = 1.70, *P* = 0.000, AHR = 0.99, *P* = 0.994) exhibited a higher risk of dysphagia before adjustment. The results are presented in Table 2.

**FIGURE 2:**
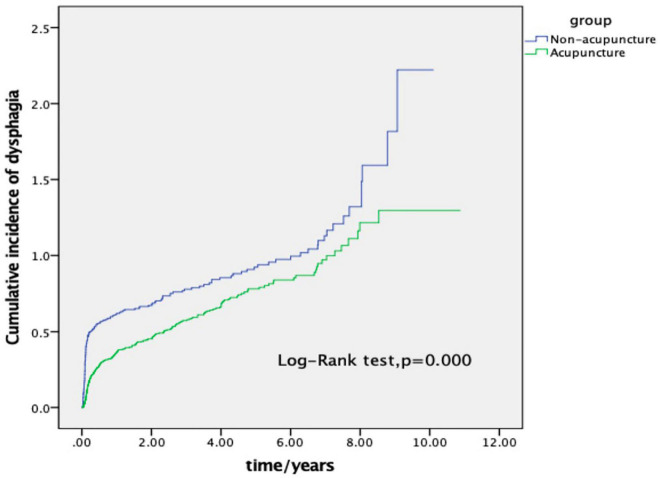
Cumulative incidence of insomnia between the acupuncture and non-acupuncture cohorts of stroke patients. As shown in the figure, the cumulative incidence of the acupuncture cohort was significantly lower than that of the non-acupuncture cohort (log-rank test, *p* = 0.000).

Regardless of age, gender, stroke location, stroke type, or baseline comorbidity, the incidence and risk ratio of dysphagia in the AP group were remarkably lower (AHR < 1) than those in the non-AP group. The overall risk of dysphagia associated with AP and non-AP in stroke patients was also determined. As shown in Table 3, multivariate stratification results indicated that patients treated with AP had a decreased risk of dysphagia as follows: men (AHR = 0.45, *P* = 0.000) or women patients (AHR = 0.33, *P* = 0.000); those aged 18–39 years (AHR = 0.20, *P* = 0.016), 40–59 years (AHR = 0.37, *P* = 0.000), 60–79 years (AHR = 0.41, *P* = 0.000), or > 80 years (AHR = 0.45, *P* = 0.000); with ischemic stroke (AHR = 0.47, *P* = 0.000), hemorrhagic stroke (AHR = 0.28, *P* = 0.000), or mixed types of stroke (AHR = 0.17, *P* = 0.000); in the brain stem (AHR = 0.41, *P* = 0.040), diencephalon (AHR = 0.13, *P* = 0.016), or with multiple partial strokes (AHR = 0.40, *P* = 0.000); with hypertension (AHR = 0.33, *P* = 0.000) or without hypertension (AHR = 0.64, *P* = 0.000); with diabetes (AHR = 0.38, *P* = 0.000) or without diabetes (AHR = 0.47, *P* = 0.000); with hyperlipidemia (AHR = 0.38, *P* = 0.000) or without hyperlipidemia (AHR = 0.46, *P* = 0.000); with mental disorder (AHR = 0.47, *P* = 0.000) or without mental disorder (AHR = 0.41, *P* = 0.000); without repeated strokes (AHR = 0.30, *P* = 0.000); with dementia (AHR = 0.50, *P* = 0.000) or without dementia (AHR = 0.40, *P* = 0.000); with Parkinson’s disease (AHR = 0.36, *P* = 0.000) or without Parkinson’s disease (AHR = 0.42, *P* = 0.000); with brain injury (AHR = 0.32, *P* = 0.000) or without brain injury (AHR = 0.44, *P* = 0.000); with lung infection (AHR = 0.29, *P* = 0.000) or without lung infection (AHR = 0.44, *P* = 0.000); and with carotid atherosclerosis (AHR = 0.34, *P* = 0.000) or without carotid atherosclerosis (AHR = 0.49, *P* = 0.000). Furthermore, the cumulative incidence of dysphagia was markedly lower in the AP group than in the non-AP group (log-rank test, *P* = 0.000, Figure 2).

**TABLE 3:** The respective hazard ratios and Incidence rates of the acupuncture and non-acupuncture cohorts before and after adjustment for gender, age, stroke type, stroke site, and baseline complications.

Variables	Acupuncture treatment						Compared with non-acupuncture users			
	No (*n* = 1,809)	Yes (*n* = 1,809)					Crude HR	*P*-value	Adjusted HR	*P*-value
	Event	Person years	IR	Event	Person years	IR	(95% CI)		(95% CI)	
**Total**	519	263,054	0.20	399	419,030	0.10	0.45 (0.39–0.51)	0.000	0.43 (0.37–0.49)	0.000
**Sex**										
Men	342	183,953	0.19	284	274,340	0.10	0.48 (0.41–0.56)	0.000	0.45 (0.38–0.53)	0.000
Women	177	79,101	0.22	115	144,690	0.08	0.38 (0.30–0.48)	0.000	0.33 (0.26–0.43)	0.000
**Age group**										
18–39	13	1,758	0.74	10	6,323	0.16	0.45 (0.19–1.04)	0.061	0.20 (0.05–0.74)	0.016
40–59	143	33,676	0.42	104	94,307	0.11	0.36 (0.28–0.47)	0.000	0.37 (0.28–0.48)	0.000
60–79	284	163,247	0.17	216	227,841	0.09	0.47 (0.39–0.56)	0.000	0.41 (0.34–0.49)	0.000
≥80	79	64,373	0.12	69	90,559	0.08	0.52 (0.37–0.71)	0.000	0.45 (0.32–0.64)	0.000
**Stroke type**										
Ischemic stroke	410	229,123	0.18	341	377,096	0.09	0.48 (0.42–0.56)	0.000	0.47 (0.40–0.54)	0.000
Hemorrhagic stroke	76	20,378	0.37	43	32,090	0.13	0.32 (0.22–0.47)	0.000	0.28 (0.18–0.43)	0.000
Both types of stroke	33	13,553	0.24	15	9,844	0.15	0.23 (0.13–0.43)	0.000	0.17 (0.08–0.35)	0.000
**Location of the stroke**										
Radiation crown	14	2,879	0.49	37	23,852	0.16	1.04 (0.55–1.96)	0.904	0.77 (0.39–1.51)	0.443
Brain stem	14	1,005	1.39	15	17,846	0.08	0.56 (0.26–1.19)	0.131	0.41 (0.18–0.96)	0.040
Diencephalon	9	4,784	0.19	8	13,115	0.06	0.35 (0.12–0.98)	0.045	0.13 (0.02–0.68)	0.016
Multiple lesions	473	253,701	0.19	335	361,200	0.09	0.43 (0.37–0.49)	0.000	0.40 (0.35–0.46)	0.000
**Baseline disease**										
**Hypertension**										
No	159	157,501	0.10	165	165,767	0.10	0.66 (0.53–0.82)	0.000	0.64 (0.51–0.81)	0.000
Yes	360	105,553	3.41	234	253,263	0.09	0.35 (0.30–0.41)	0.000	0.33 (0.28–0.40)	0.000
**Diabetes**										
No	269	108,600	0.25	225	210,666	0.11	0.48 (0.40–0.57)	0.000	0.47 (0.39–0.56)	0.000
Yes	250	154,454	0.16	174	208,364	0.08	0.42 (0.34–0.51)	0.000	0.38 (0.31–0.46)	0.000
**Hyperlipidemia**										
No	280	160,276	0.16	225	219,575	0.10	0.47 (0.39–0.56)	0.000	0.46 (0.38–0.55)	0.000
Yes	239	102,778	0.23	174	199,455	0.09	0.42 (0.34–0.51)	0.000	0.38 (0.31–0.47)	0.000
**Mental disorder**										
No	423	192,394	0.22	307	311,266	0.10	0.43 (0.37–0.49)	0.000	0.41 (0.35–0.47)	0.000
Yes	96	70,660	0.14	92	107,764	0.09	0.52 (0.39–0.70)	0.000	0.47 (0.35–0.64)	0.000
**Repeated strokes**										
No	343	30,238	1.13	207	105,315	0.20	0.31 (0.26–0.37)	0.000	0.30 (0.25–0.36)	0.000
Yes	176	232,816	0.08	192	313,715	0.06	0.90 (0.73–1.11)	0.321	0.87 (0.70–1.07)	0.176
**Dementia**										
No	376	190,734	0.20	269	245,592	0.11	0.41 (0.35–0.49)	0.000	0.40 (0.34–0.47)	0.000
Yes	143	72,320	0.20	130	173,438	0.07	0.55 (0.43–0.70)	0.000	0.50 (0.39–0.65)	0.000
**Parkinson’s**										
No	443	215,980	0.21	352	348,148	0.10	0.45 (0.39–0.52)	0.000	0.42 (0.36–0.48)	0.000
Yes	76	47,074	0.16	47	70,882	0.07	0.41 (0.29–0.60)	0.000	0.36 (0.24–0.54)	0.000
**Brain injury**										
No	458	239,569	0.19	356	377,046	0.09	0.45 (0.39–0.51)	0.000	0.44 (0.38–0.51)	0.000
Yes	61	23,485	0.26	43	41,984	0.10	0.41 (0.28–0.62)	0.000	0.32 (0.20–0.49)	0.000
**Lung infection**										
No	441	239,183	0.18	347	353,052	0.10	0.45 (0.39–0.52)	0.000	0.44 (0.38–0.51)	0.000
Yes	78	23,871	0.33	52	65,978	0.08	0.41 (0.29–0.59)	0.000	0.29 (0.20–0.42)	0.000
**Carotid atherosclerosis**										
No	295	178,802	0.16	234	227,142	0.10	0.49 (0.41–0.58)	0.000	0.49 (0.41–0.59)	0.000
Yes	224	84,252	0.27	165	191,888	0.09	0.38 (0.31–0.47)	0.000	0.34 (0.28–0.42)	0.000

*IR, incidence rates, per 1,000 person-years; HR, hazard ratio; CI, confidence interval.*

*Adjusted HR: adjusted for age, gender, diabetes, sex, age, stroke type, stroke location, and baseline disease: hypertension, diabetes, hyperlipidemia, mental disorder, repeated strokes, rementia, Parkinson’s, brain Injury, lung infection in Cox proportional hazards regression.*

## Discussion

The results showed that AP treatment can attenuated the risk of dysphagia in stroke patients of different ages, genders, stroke types, stroke locations, and baseline comorbidities. In addition to the analysis of conventional risk factors (e.g., diabetes, hypertension, dyslipidemia, lung infection, and brain injury), we also included current hotspot research factors such as dementia, Parkinson’s disease, and psychological disorders to examine the relationships between these diseases and PSD. Because research on these factors is still lacking, we analyzed, summarized, and classified these factors, and evaluated their impact on PSD to obtain more information on risk factors for PSD.

In our study, most of the patients with PSD were in the 60–79 age group, which is in good agreement with previous findings (Mann and Hankey, 2001). This trend is probably related to the more complicated pathophysiological changes that occur in elderly stroke patients. For example, the total muscle volume of elderly patients decreases, especially swallowing muscles that shrink with age, along with a decrease in tongue pressure (Sporns et al., 2017). In addition, elderly people often have other neurological diseases (e.g., Parkinson’s disease and dementia) that can also cause swallowing problems. The combination of these mechanisms working together results in a high prevalence of PSD in elderly stroke patients (Warnecke et al., 2019). Regarding the location of the stroke lesions, our study showed a greater number of patients had multiple strokes, which increased the risk of developing PSD compared with that of other stroke locations (CHR = 4.29, *P* = 0.012; AHR = 3.39, *P* = 0.035). Stroke with multiple lesions indicates that the severity of stroke is higher. An increase in the number of lesions causes greater damage to the brain and a wider range of nerve damage, resulting in a high incidence of dysphagia. Evidence from MRI images also shows a significant positive correlation between the severity of dysphagia and the number of lesions (Otto et al., 2016), which results in a high incidence of PSD. Our study also showed that when the NIHSS cut-off value was ≥12, the severity of stroke was an important predictor of persistent dysphagia patterns (Toscano et al., 2015).

Regarding baseline diseases, in addition to common risk factors such as diabetes and hypertension that are recognized by most researchers, we focused on the impact of the following factors on the development of PSD. Dementia presented a clear risk in this study as it increased the risk of PSD (AHR = 1.62, *P* = 0.000). The contribution of dementia to the risk of dysphagia lies in its impact on swallowing which is related to the partial overlap of neuroanatomy with cognitive cortical areas (Somasundaram et al., 2014). Some researchers have also proposed that poor cognitive function weakens the swallowing ability by affecting the patients’ visual attention and execution ability (Jo et al., 2017). Patients with dementia are also prone to aspiration, with aspiration-induced pneumonia being the most frequent cause of death in these patients (Castagna et al., 2019). In view of these connections between dysphagia and cognitive impairment, related pathological models have indicated that combining cognition and dysphagia as an intermediary of treatment might be beneficial for the treatment of dysphagia (Ebrahimian Dehaghani et al., 2019).

Pulmonary infections also presented as corresponding risks in the study (AHR = 1.22, *P* = 0.044). Dysphagia is the main risk factor for pneumonia in the first week after a stroke (Brogan et al., 2014). After a stroke, the risk of a respiratory tract infection associated with dysphagia is 10 times higher than that in normal patients, and the risk of death is 18 times higher than that of healthy patients (Sala et al., 1998). Dysphagia and lung infection are mutual risk factors for post-stroke complications, with pulmonary infections also being the greatest hidden danger of dysphagia after a stroke (Martino et al., 2005). Patients with dysphagia after a stroke are more likely to be administered antibiotics, although our scientific understanding and effective use of antibiotics in these cases remain poor (Finniss et al., 2021). Future research should therefore focus on the use of standardized antibiotic therapy.

Research on the relationship between traumatic brain injury and PSD is also insufficient. Although our research showed that traumatic brain injury is a risk factor for PSD (AHR = 1.40, *P* = 0.002), the explanation of its effect on PSD is complicated. Brain injury such as that caused by Parkinson’s disease or dementia is accompanied by nerve damage, with Parkinson’s disease or dementia induced by brain injury which causes PSD. These disorders often interact with each other, with mutual causes and effects (Florea et al., 2020), and therefore more detailed research is needed to determine the pathological relationship between brain injury, Parkinson’s disease and dementia. However, after adjustment of the data, we showed the differences were not obvious and that the risk was variable, possibly because traumatized brains may be influenced by other variables under certain conditions. Therefore, we suggest that researchers should assess these factors in future studies and attempt to exclude the impact of other covariates.

The impact of Parkinson’s disease on the risk of developing PSD raises interesting questions in this study. Before adjusting for the influence of other variables, Parkinson’s disease was associated with a degree of risk for the development of PSD (CHR = 1.70, *P* = 0.000). However, the difference was not significant after adjustment, indicating that the risk is variable. This may be because the fact that these factors are influenced by other variables under certain conditions. However, we still tend to consider Parkinson’s disease as a risk factor for PSD. Studies have shown that elderly stroke patients who are also taking levodopa drugs have an increased risk of 50–77% to develop swallowing disorders (González-Fernández et al., 2013), which may indicate progression of the disease. The pathogenic mechanism is related to muscle stiffness and motor retardation in patients with Parkinson’s disease (Umemoto and Furuya, 2020), and may also be related to poor coordination of the muscles of the oropharynx in these patients (Claus et al., 2020). In fact, patients with advanced Parkinson’s disease are often tube fed, which may conceal the presence of dysphagia, thereby underestimating the impact of Parkinson’s disease on dysphagia (Takizawa et al., 2016). This possibility may also help to explain the vulnerability of patients with Parkinson’s disease in certain circumstances due to the influence of other variables. We, therefore, recommend that researchers conduct special studies on these relationships and attempt to eliminate the impact of other factors.

Regarding the baseline diseases on PSD, the impact of mental disorders on PSD was the main focus of our research. Depression and anxiety are common in stroke patients. Swallowing dysfunction after stroke is a form of post-stroke stress, which itself contains mental stimulation and emotional stimulation. These stimulations will cause the corresponding neuroendocrine changes in the body (Xi et al., 2021). For example, most stroke patients tend to show pessimism such as indifferent expressions and refusal to communicate. These negative emotions ultimately affect the autonomy of patients rehabilitating after a stroke, and may even affect objective rehabilitation methods provided by the hospital, ultimately weakening the efficacy of these various rehabilitation methods. Therefore, dysphagia and depression after a stroke affect each other and are closely related. In our study, psychological disorders exhibited a higher risk before and after adjustment for covariates (CHR = 1.99, *P* = 0.000; AHR = 1.55, *P* = 0.000). In addition, patients with PSD also suffer from dysarthria and aphasia. A previous study reported that the incidence of dysphagia, dysarthria, and aphasia were 32, 26, and 16%, respectively. These diseases are commonly characterized by stroke speech pathology and are all likely to induce mental disorders (Stipancic et al., 2019). At present, the increased attention on the topic of post-stroke depression is also inextricably linked to the above-mentioned diseases (Leentjens et al., 2006). However, there has been little attention has been paid to the impact of mental disorders on PSD, and only a few researchers have realized that mental disorders are negatively related to dysphagia (Kim et al., 2020). Evidence from functional magnetic resonance imaging suggests that the pathogenesis of dysphagia is not only related to the brain’s motor region, but that affective network regions are also related to dysphagia in stroke patients. These relationships are mainly affected by emotional changes. The patient’s psychological state, such as anxiety and depression acts on the emotional network area by stimulating emotions (Liu et al., 2017). Intermittent dysphagia is related to anxiety, whereas progressive dysphagia is related to depression (Eslick and Talley, 2008). These associations increase our understanding of the pathogenesis of dysphagia after stroke and should lead to the development of more targeted clinical treatments.

The patient information in our clinical study was collected retrospectively, and we observed that the AP prescription was not standardized for all patients, although the main AP points were the same. The most commonly used AP points included Baihui (GV 20), Shenting (GV 23), Fengfu (GV 16), Yuye (EX-HN 13), Jinjin (EX-HN 12), Lianquan (CV 23), Zhaohai (KI 6), and Lieque (LU 7). The roles of these AP points have been studied previously, and their therapeutic mechanism has been investigated (Li et al., 2019).

Therefore, although the AP prescription included the AP points directly related to the PSD pathology (Fengfu, Lianquan, Lieque, and Zhaohai), it also included the composition of acupoints (Baihui and Shenting) that regulate the mental activity and mental state of the human body from an overall macroscopic perspective. Baihui belongs to the Du meridian. Traditional Chinese medicine (TCM) considers that the Du meridian is related closely to neurological activities. Baihui was defined as “the brain the home of the mind” according to the “Compendium of Materia Medica.” AP at acupoints on the Du meridian is used to treat mental illnesses. Fengfu belongs to the Du meridian and is related closely to the mental activity of the human body. In addition, the Fengfu acupoint is located beside the brainstem and cerebellum, close to the lesions that cause swallowing dysfunction after a stroke. Stimulating Fengfu acupoints adjacent to the lesion where the AP points pass can therefore have a therapeutic role and help to restore swallowing function. Studies have also shown that electrical AP stimulates the Fengfu acupoint to promotes the recovery of cranial nerves by down-regulating the neurotoxin mediated by S100β (Lu et al., 2010). Lianquan belongs to the Ren meridian. The AP point is located in the neck, between the thyroid cartilage and the hyoid bone, with the hypoglossal nerve being distributed deep in the tissues. AP at this point strengthens the stimulation of the hypoglossal nerve, enhances sensitivity of the nerve, and improves swallowing function. The ancient book “Experience on Acupuncture and Moxibustion Therapy” records that the “Lianquan acupoint mainly treats sublingual swelling and unspeakable, tongue salivation” (Li et al., 2020). The Lieque and Zhaohai acupoints belong to the eight convergent points. Lieque intersects with the Ren meridian, whereas Zhaohai connects with the Yinqiao channel. The circulation routes of the two meridians, the Ren meridian and the Yinqiao channel, both pass through the chest, lungs, diaphragm, and throat, and therefore AP at these two acupoints can treat diseases related to swallowing. These acupoints prescriptions are an important part of “mind-regulating AP” in traditional Chinese medicine, which focuses on adjusting the human body’s mental activities and the balance of qi, blood, yin, and yang to achieve the effect of curing diseases. “Su Wen” states that, “Ancient people…if the body and spirit are harmoniously combined into one, the quality of life and life span of the person will be high.” The so-called “body and spirit are combined into one,” that is, only when the body and spirit are harmoniously combined can the human body be disease-free. “Su Wen” also pointed out that “tranquil and emptiness, the true qi will follow it, spirit inward, and the disease will not occur.” This indicates that maintaining a stable and healthy mentality has an important role in preventing the occurrence of diseases. At present, regarding “mind-regulating AP therapy,” studies have proposed that this is a benign pre-stress hypothesis mechanism that explains the basis of treatment (Li, 2003). However, the mechanism of AP is far more complicated than this, because it involves neuroendocrine, cardiovascular, metabolic, and even genetic theories (Chavez et al., 2017). In the future, precise research on AP should be carried out to provide a theoretical basis for its further promotion.

The swallowing function involves multiple parts of the brain including the brainstem, basal ganglia, corona, cerebellum, and the cerebral cortex (Zald and Pardo, 1999). PSD is caused by damage to the cortex and subcortical structures and strokes that occur in the dominant hemisphere that control swallowing function are more likely to induce damage to this function. The mechanism of AP treatment of PSD is related to the following. AP excites the paralyzed peripheral motor fibers of the medulla oblongata, restores voluntary movement of the glossopharynx, strengthens the blood oxygen supply of the diseased area, improves blood circulation in the motor function area of the cerebral cortex, and promotes the establishment of peripheral blood vessel collateral circulation and nerve cell activation, while strengthening the coordination and compensation between the functional areas of the cortex (Zhang et al., 2016; Li et al., 2017; Chang C. C. et al., 2018). Cranial diffusion tensor imaging (DTI) and the gold standard TV X-ray swallowing function (VFSS) for the diagnosis of dysphagia also show that AP can reduce the passage time of the oral phase and the delay time of the pharyngeal phase, as well as improve the drinking water test and related scale scores, which promotes the recovery from dysphagia after stroke (Zhang et al., 2018). A rat model of dysphagia after stroke suggested that AP protected neurons in the hypoglossal nerve nucleus in the medulla oblongata swallowing center, reducing the contraction threshold strength of the tongue muscle after hypoglossal nerve injury, and improving the hypoglossal nerve-glossal muscle conduction system (Jian et al., 2019). AP on the unaffected cerebral hemispheres to enhance the stimulation of the medullary motor neurons to the pharynx, improved the compensatory ability of the central swallowing reflex, and reduced the symptoms of dysphagia (Li et al., 2019). Therefore, AP not only enhances the stimulation of the medulla oblongata swallowing center, but also strengthens the surrounding muscles used for swallowing.

In recent years, AP has become widely used for the preventive treatment of numerous diseases, such as the prevention of post-stroke pulmonary infection (Chang C. C. et al., 2018), post-stroke dementia (Shih et al., 2017), post-stroke epilepsy (Weng et al., 2016), coronary heart disease related to cardiovascular disease (Wu et al., 2018), pain-related trigeminal neuralgia, fibromyalgia syndrome, and neuralgia associated with herpes zoster sequelae (Huang et al., 2020; Liao et al., 2020b; Ruengwongroj et al., 2020). It is also widely used in the management of sub-health (Chang Q. Y. et al., 2018; Chen et al., 2019). These are high-incidence and difficult diseases of common concern around the world and are examples where the concept of taking precautions before getting sick are applicable. The above-mentioned research shows that AP can prevent and treat diseases of different systems. In addition, AP can significantly reduce the medical expenditure of patients and relieve the economic burden on both patients and the government (Liao et al., 2020a). In general, AP has a wealth of historical and practical data, a wide range of active mechanisms, high social recognition, a wide range of diseases that can be treated, and is safe and relatively economical. It is therefore worthy of clinicians’ attention, further use, and promotion. Future research should focus on the pathophysiology and mechanisms related to the AP treatment of different diseases, which will make the clinical application of AP more scientific.

## Limitations

This study had some limitations. First, this was a single-center study carried out at the First Affiliated Hospital of GUCM. This hospital mainly uses TCM practices such as AP and herbal treatments. Therefore, it is necessary to conduct multi-center research in the near future. Second, our findings demonstrated that AP had preventive therapeutic advantages for the treatment of PSD. However, we did not classify the severity of PSD, nor did we stage the dysphagia by considering the pathological characteristics of the condition, and therefore the selection of AP points could be different. To accurately guide the clinical use of AP, a more detailed description should be provided, such as an AP plan, specific operation method, and selection of times for treatment. Lastly, to draw evidence-based conclusions, additional retrospective studies with larger sample sizes are warranted in the future.

## Data Availability Statement

The raw data supporting the conclusions of this article will be made available by the authors, without undue reservation.

## Ethics Statement

The studies involving human participants were reviewed and approved by the First Affiliated Hospital of Guangzhou University of Chinese Medicine. Written informed consent for participation was not required for this study in accordance with the national legislation and the institutional requirements.

## Author Contributions

HZ and XQ conceived the initial research direction and drafted the manuscript XQ, X-JY, S-NH, Y-YW, T-SL, and Z-JO was responsible for data collection, inputted and sorted during the whole process. XQ and X-JY was responsible for verifying and checking the correctness of the data and also responsible for data statistics. HZ was responsible for the guidance and suggestions of the entire research process. All the above authors have contributed to this research and completely agreed for publication.

## Conflict of Interest

The authors declare that the research was conducted in the absence of any commercial or financial relationships that could be construed as a potential conflict of interest.

## Publisher’s Note

All claims expressed in this article are solely those of the authors and do not necessarily represent those of their affiliated organizations, or those of the publisher, the editors and the reviewers. Any product that may be evaluated in this article, or claim that may be made by its manufacturer, is not guaranteed or endorsed by the publisher.
